# How do red foxes (*Vulpes vulpes*) explore their environment? Characteristics of movement patterns in time and space

**DOI:** 10.1186/s40462-024-00526-1

**Published:** 2025-01-16

**Authors:** Felicitas Oehler, Robert Hagen, Klaus Hackländer, Zea Walton, Kumar Ashish, Janosch Arnold

**Affiliations:** 1 Wildlife Research Unit Baden-Württemberg, LAZBW, Atzenberger Weg 99, 88326 Aulendorf, Germany; 2 Institute of Wildlife Biology and Game Management, BOKU University, Vienna, Austria; 3 Deutsche Wildtier Stiftung (German Wildlife Foundation), Hamburg, Germany; 4 Department of Forestry and Wildlife Management, Faculty of Applied Ecology and Agricultural Sciences, Inland Norway University of Applied Sciences, Evenstad, Norway; 5 Department for the Ecology of Animal Societies, Max Planck Institute of Animal Behavior, 78467 Constance, Germany; 6 Salim Ali Centre for Ornithology and Natural History, Coimbatore, India; 7 Department of Biometry and Environmental System Analysis, University of Freiburg, Freiburg, Germany

**Keywords:** Movement ecology, Habitat utilization, Exploration, Dispersal, Random forest model

## Abstract

**Background:**

Many animals must adapt their movements to different conditions encountered during different life phases, such as when exploring extraterritorial areas for dispersal, foraging or breeding. To better understand how animals move in different movement phases, we asked whether movement patterns differ between one way directed movements, such as during the transient phase of dispersal or two way exploratory-like movements such as during extraterritorial excursions or stationary movements.

**Methods:**

We GPS collared red foxes in a rural area in southern Germany between 2020 and 2023. Using a random forest model, we analyzed different movement parameters, habitat features—for example landclasses and distances to linear structures—and time variables (season and time of day) within red fox exploratory, transient and stationary movement phases to characterize phase specific movement patterns and to investigate the influence of different variables on classifying the movement phases.

**Results:**

According to the classification model, the movement patterns in the different phases were characterized most strongly by the variables persistence velocity, season, step length and distance to linear structures. In extraterritorial areas, red foxes either moved straight with high persistence velocity, close to anthropogenic linear structures during transient movements, or more tortuously containing a higher variance in turning angles and a decrease in persistence velocity during exploratory-like movements. Transient movements mainly took place during autumn, whereas exploratory-like movements were mainly conducted during winter and spring.

**Conclusion:**

Movement patterns of red foxes differ between transient, exploratory and stationary phases, reflecting displacement, searching and resident movement strategies. Our results signify the importance of the combined effect of using movement, habitat and time variables together in analyzing movement phases. High movement variability may allow red foxes to navigate in extraterritorial areas efficiently and to adapt to different environmental and behavioral conditions.

## Background

Animals may exhibit large-scale seasonal movements in response to changing resource availability or environmental conditions such as during migration [1], or they may move locally for daily foraging within a home range [2–5]. Such movements may occur along known routes or paths [6, 7]. However, sometimes animal movements may be exploratory in nature i.e. outside of a known area or home range [8], such as during natal dispersal when searching for a place to settle or for resources or mating opportunities in extraterritorial or completely novel areas [5]. Thus, movements can be differentiated into stationary or extraterritorial movements. Extraterritorial movements are risky as animals might face rather unpredictable encounters with vehicles [9, 10], territorial individuals [11], or predators [12]. They might also have to search long or far to find food or shelter, resulting in an increase in energy consumption [13]. Yet, despite the risks and the costs associated with movements outside a home range, such movements are crucial to gather information [14, 15], to reach novel areas and to expand or adjust home range areas to more favorable habitats, resulting in increased fitness [16]. As animals cannot rely on their cognitive maps when navigating these novel areas, they must instead actively sample their environment by sensory information [17], which will influence their movement patterns [2, 18]. Thus, animal movement patterns in extraterritorial areas are likely to differ from movements in settled areas, hereafter defined as within home range movements (stationary phase) [19, 20].

Extraterritorial movements can be differentiated as exploratory movements outside settled areas including a return (exploratory phase) or as one-way movements, where individuals leave a settled area to move to a new area, as during dispersal (transient phase). Roshier et al. [8] showed that individuals often move fast and directed when outside their home ranges to minimize risk. These fast and directed movements are designed for displacement [21] and mainly observed for movements between patches, which reflects transient movements [22]. This movement strategy enhances energy savings during the transient phase of a dispersal event [23], while also allowing individuals to quickly pass occupied territories or risky areas, which has an influence on their movement pattern, even without direct encounters of the territory holder, for example a conspecific [24].

During the exploratory phase, on the other hand, movement patterns may be characterized by strategies to collect new information about the environment, resources and conspecifics [25]. This can reflect a searching behaviour, where individuals search for mates or look for foraging opportunities. Searching movements might result in high levels of returning to known areas [21], and include many directional changes, slower movements and smaller step lengths, similar to that represented by Lévy walks or correlated random walks [26]. During the exploratory phase, animals may move linearly until they locate a resource, at which time they may continue with an area restricted search, changing their movement pattern to a more tortuous path, until the resource is depleted [27]. Exploratory movements might also be conducted for information gathering or to explore the area before dispersal [28, 29].

Movement patterns are further influenced by external factors. In the surrounding environment, animals select for their optimal habitat to maximize survival and fitness [30–34]. Different habitat types, habitat features and structures promote different movement patterns [19, 31, 35]. Linear structures facilitate faster movement, as shown for dispersing male brown bears (*Ursus arctos*), who moved faster when closer to small forestry roads and larger public roads in Sweden [36]. Habitat selection may also be influenced by temporal patterns such as diurnality or seasonality. For example, deer species show diurnal habitat preferences where they select more forested/covered areas during day than during night, when they prefer open habitats [37, 38]. Season can further influence movement patterns, since resources or density capacities in space might change between seasons [39, 40]. Thus, movement patterns in different movement phases might be influenced by habitat features and timing. Dispersing individuals may select for habitat that facilitates more direct movement, while during exploratory-like movements animals may prefer to move in open areas to enhance searching efficiency. Taking movement, habitat and timing variables together into account, we want to analyse in this study how these variables characterize the movement patterns in the movement phases exploratory (including exploratory-like movements), transient and stationary.

Red foxes (*Vulpes vulpes*) are one of the most widely distributed terrestrial species across the globe, representing a very adaptable mammal species [41–43]. As a generalist, red foxes prefer spatially heterogeneous landscapes with a high diversity in habitat types, thus a high distribution of vegetation features, allowing them to utilize a wide variety of prey, food resources and shelter opportunities [44, 45]. Subadult red foxes disperse during autumn [46]. In highly fragmented landscapes, red foxes are known to follow linear structures [47].

In this study, we use the red fox as our model species to investigate movement patterns, habitat preferences and interactions between timing, movement and habitat features during exploratory, transient and stationary phases. Investigating how red foxes move in extraterritorial areas will assist in improving our understanding of the adaptability and range expansion capabilities of red foxes, which is important due to its potential for disease spread [48] and its impact on other species through predation and competition [49, 50]. Specifically, we sought to understand how different movement, habitat, and temporal (e.g., diurnality and seasonality) variables described the exploratory-like, transient and stationary movement phases of red foxes. To do this, we used data from GPS-collared red foxes in Germany. We analyzed red fox movement patterns in a landscape heavily dominated and altered by human land use to explore how foxes moved during these different movement phases. We predicted that

1. during transient movement phase, movement patterns will be characterized by more directed movements (persistence velocity, step length and turning angle), compared to stationary and exploratory phases;
2. during exploratory phase consisting of exploratory-like movements, movement patterns will be characterized by a smaller step length and a more tortuous path, compared to transient and stationary phases;
3. seasonal differences will occur between transient and exploratory-like movements. Thus, transient movements will occur more frequently during autumn, when subadult individuals are known to disperse and exploratory movements will occur more frequently during spring, when individuals search for resources.

## Material and methods

The presented study was conducted in a rural area in South Germany (47° 51′ 18.0″ N 9° 32′ 11.8″ E) comprising 142,400 hectares. The region is located in the pre-Alpine hillscape and moor landscape, consisting of small structured cultivated agricultural or greenland fields and patterns of forest with spruce (*Picea abies*) as main tree species, representing a heterogeneous landscape [51]. Agricultural land use is mainly arable farming, grassland and livestock farming [52, 53]. The annual average temperature is 8.5 °C and the average annual precipitation amounts to 941 mm [54]. We equipped 26 red foxes [19 males: 4 adults, 15 subadults; 7 females: 2 adults, 5 subadults, Table 2 in appendix] with GPS-collars (‘Collar 1C’; 170 g, e-obs, Grünwald, Germany and ‘Tellus ultra light’, 213 g, Followit, Lindesberg, Sweden) between October 2020 and March 2023. Red foxes were trapped in live traps (wooden box traps, concrete pipes) between 1st September and 15th February each year, to ensure that no adult animal was kept away from offspring during denning season and that subadults were large enough to be equipped with a GPS-collar. Traps were equipped with a remote alarm system (Trapmaster, EPV Electronics GmbH, Germany) sending a message via mobile net as soon as the trap closed. Each closed trap was checked immediately, latest after dawn. Bycatches were released. Red fox individuals were immobilized as described by [55]. Applied dose of anesthetics composed of 0.03 ml/kg ketamine (Anesketin, Dechra) and 0.08 ml/kg medetomidine (Sedator®, Dechra) was injected intramuscularly. Additionally, sex, age, body weight and size was noted (see Table 2). Age was determined based on the state of the teeth and defined as subadult (< 1 year) and adult (> 1 year) [56–59]. After deployment of the GPS-collars, the antagonist Atipamezole (Atipam®; Dechra) of the same volume as the Sedator was injected. All procedures were accepted by the ‘Regierungspräsidium Tübingen’ under the animal protection law, registered as LAZ4/19G and supervised by veterinarians.

Collars were programmed to collect a GPS location by activity of the collared animal every 10 min and every 1 h when inactive (e-obs) or every 20 min during night and every 3 h during day (Followit). Thus, the time between two fixes of the e-obs collars depended on the activity of the individual. We collected 248,338 GPS points in total. We first removed spatial outliers using QGIS version 3.22.12 [60] (for detailed information see appendix ‘Outlier removal’) and excluded all GPS locations taken within the first 24 h to exclude trapping bias [61]. Four individuals that collected less than one month GPS data were excluded from further analyses (Table 2). This resulted in 22 individuals for further analysis. For each individual, we calculated movement tracks, where each pair of subsequent locations lies within a temporal range (dt) between 10 and 60 min, using the package amt [62]. We decided on an interval for dt between 10 and 60 min as 10 min corresponds to the minimal fix interval and 60 min minimized the total number of locations discarded. If the temporal distance exceeded the threshold of 60 min the next GPS location became the beginning of a sequence of subsequent locations within the dt. However, in the case that dt to the following GPS-location also exceeded 60 min, these locations were discarded. Dt for less than 10% of all subsequent locations exceeded 60 min. This left 120,359 GPS points (see appendix Table 2 for further details with time of data collection per individual). Data analysis was conducted with R version 4.1.3 [63].

**Fig. 1:**
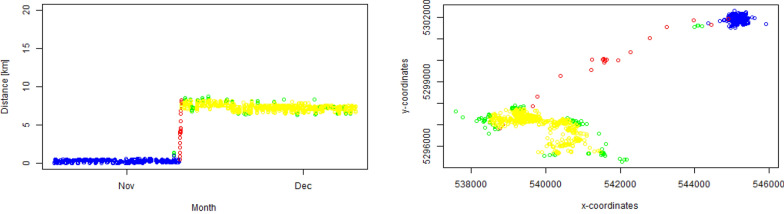
Visualization of GPS location data showing movement parameters of a single red fox male. The left box shows a time series of net squared displacement (NSD) from an initial starting position and the right box the x and y coordinates (in UTMS). The stationary phase (S_1_ and S_2_) is shown in blue (S_1_) and yellow (S_2_), the transient phase in red and exploratory phase in green. The x and y coordinates of all individuals in the stationary and transient phases are visualized in Fig. 5

To examine exploratory-like and transient movement patterns and habitat preferences of red foxes, we separated the movement paths of all individuals into exploratory-like, transient and stationary movement phases (Fig. 1, Fig. 5). Identifying transient and stationary phases in mammals using movement data remains very challenging [64–66]. The variability of movement data [64] makes it nearly impossible that a single method identifies correctly all possible segmentation parts in a movement path and thus reliably identifies transient and stationary phases [67]. For assigning the transient (T) and stationary phases (S_1_, S_2_) (Fig. 5) we used the classification of Oehler et al. [68] that is based on (a) the detection of early warning signals [69, 70] using times series of the net squared displacement (NSD—[71]) and (b) the evaluation of early warning signals via the application of a k-means cluster algorithm provided via the R-package marcher [72]. According to Oehler et al. [68], eight individuals were classified as dispersers. We classified the transient phase as the movement phase in a dispersal event, which began with leaving a stationary state (S_1_) and finished when arriving at a second stationary state (S_2_). The stationary states belonged to the stationary phase. The analysis by Oehler et al. [68] suggested that three other individuals were probably caught during the transient phase and one individual stopped collecting data probably during the transient phase. Since we were able to identify two distinct spatial clusters for those four individuals we used the time from beginning of data collection until start of S_2_ or end of S_1_ until stop of data collection as transient phase. Thus, eight dispersers and four individuals caught during dispersal, resulting in twelve out of 26 red fox individuals experiencing transient and stationary movement phases during data collection. We continued our analysis with these twelve individuals. We further defined a GPS point as exploratory-like when the x and y coordinates of the GPS point or the distance to the first location (net squared displacement, NSD) was outside of the 98.5% quantile or 1.5% quantile during the stationary phases (Fig. 1). For detailed explanation see appendix 'Classification of exploratory movements'. Thus, we ensured that the movement trajectories were outside of the stationary area (S_1_ and S_2_) but included a return to the stationary area. Consequently, the timing of the exploratory GPS points occurred within the timespan of the stationary phase. For the calculation of the x/y and NSD distribution in S_2_ we used the first four weeks of location data, since data collection was sometimes multiple months long in S_2_. We further included the condition that every point in S_1_ was stationary, (1) when the point was less than 1 km away from the trapping point and (2) when during the transient phase the animal returned to the trapping location (NSD < 1 km). This resulted in segmented movement tracks of each individual into stationary, transient and exploratory-like movement phases (Fig. 1, Fig. 5). We consider S_1_ and S_2_ together in the stationary phase.

To identify movement patterns within the classified stationary, exploratory-like and transient movement phases, we analyzed influences of movement variables, habitat features and timing on the movement patterns in the specific phases. We calculated turning angle (ta), step length (sl) and persistence velocity (pv) using the movement tracks of the twelve individuals with the amt package [62]. Step length neither increased nor decreased systematically for dt ranging between 10 and 60 min (Fig. 4). The variable pv was calculated as speed_i_*cos(ta_i_) for each individual i. These movement variables have been used by other studies to identify movement [67, 73, 74], and thus seemed appropriate to reflect movement patterns in the movement phases. The variable turning angle reflects how straight or tortuous the path might be, step length shows the distance moved and speed is the distance traveled in a given time interval between two relocations [67, 73]. Using the variable speed we further calculated the persistence velocity (pv = speed_ti_*cos(ta_ti_), where t_i_ is each timestamp in the timeseries data), the tendency of a movement to persist in a certain direction [67, 73, 74]. To link movement data to habitat features, we used satellite data for landscape classification derived from ESA world cover maps 2021 [75], resolution 10 m × 10 m. The composition of the vegetation in the study area is made up by 41.5% forest, 25.6% Greenland, 25.5% agricultural fields, 5.4% anthropogenic structures and 2% belonging to other vegetation types such as water, wetland and sparse vegetation (100% minimum convex polygon (MCP) area plus 7 km buffer). The number of GPS points per habitat class is shown in Table 3 in the appendix. Additionally, we used a layer for road and trail networks and the river system [76], to calculate the distances to the features in QGIS (GDAL Proximity) with 1 m × 1 m resolution. The variable linear anthropogenic structures included the minimum distance to either state roads, federal highways or railways, combining the larger linear structures into one variable. The following variables, listed in Table 1, were extracted from the landscape maps:

**Table 1:** Land classifications (n = 7) according to ESA world cover maps 2021 [76] and distance variables (n = 5) reflecting the Euclidian distance to linear structures or flowing waters, conservation areas, district or agricultural roads

Land classification types (ESA): 1. Tree cover 2. Greenland 3. Agricultural field 4. Settlement 5. Sparse Vegetation 6. Water 7. Wetland	Distance variables: 1. Distance to flowing waters 2. Distance to protected areas 3. Distance to district road 4. Distance to agricultural road 5. Linear anthropogenic structures: ✓ Distance to state roads ✓ Distance to federal highways ✓ Distance to railways

To identify co-linearity of habitat variables, we used the Spearman correlation coefficient. We did not exclude any habitat layer, since all correlation values were smaller r < 0.4, indicating a low correlation between each other [77]. Afterwards, we extracted the appropriate habitat covariates at the end of each movement step using amt package. To include time variables, since dispersal movements are typically undertaken by subadults during autumn, we included the variable season (spring: March, April, May, summer: June, July, August, autumn: September, October, November, winter: December, January, February) in the analysis. We further included a time of day variable (day/night) to test if one movement phase is more likely during daylight or nighttime hours, while 16% of the GPS fixes were collected during the day and 84% during the night.

To analyze which variables are most predictive of the stationary, transient and exploratory phases, we conducted a classification based random forest model [78]. Relationships between movement patterns and habitat features or timing are intricate and can be nonlinear in nature, therefore, we applied a random forest model using randomForest package in R [78]. Random forest models are non-linear, non-parametric and do not require data assumptions in variable distributions. Furthermore, random forest models provide high accuracy in predictions [79] and it is possible to use all variables together in one model to evaluate the relative importance of different variables and how the variables’ values differ between the movement phases. However, a random forest model is vulnerable to imbalanced data with feature analysis. Our data was class imbalanced with a different number of locations per phase, where the stationary phase was the majority class (Number of GPS points per phase: stationary = 32,246; transient = 2372; exploratory = 7008). To minimize the effect of the minority class in the model, since a random forest is not able to account for differences in the sampling regime according to individuals, we selected ten steps per individual per phase and bootstrapped the data 120 times with replacement to reach a distribution of 1,200 data points per individual and per phase. Even though random forests automatically sample the data used for each tree with replacement, these techniques did not account for imbalanced data due to a different number of GPS-locations per individual and phase. Our objective was a balanced data set according to both the movement phase and the individual. With this dataset, including data from all twelve red foxes, we conducted a random forest model with 300 trees, using our movement, habitat and timing variables as predictor variables. This included in total eleven variables: landclass, distances to different habitat types (see Table 1), movement parameters (sl, ta, pv), season and time of day, and movement phases containing three classes: ‘exploratory’, ‘transient’ and ‘stationary’ as target variable. Three hundred trees seemed appropriate, as testing showed that the out of bag (OOB) error fluctuated around the same value while adding additional trees after 150 trees. Random forest is an ensemble model consisting of multiple decision trees, each trained on a different subset of the input data, drawn through bootstrapping. The observations not used for training a particular tree are referred to as OOB data for that tree. The model's error rate was calculated by validating predictions from each of the 150 trees on their respective OOB data. In this random forest model, each tree was trained on approximately 64% of the input observations, while about 36% of the observations were left out as OOB data for model validation. Additionally, we applied statistical methods to calculate class-specific error rates, providing deeper insights into the model's accuracy. As splitting criterion at each node, we used three variables randomly sampled from the predictor variables (eleven variables, which is the square root of the number of predictor variables). We combined five random forest objects into one forest model, which had in the end 1,500 trees to exclude the chance of a bias by subsampling our original data. We calculated the minimal depth of the predictor variables of the random forest by using the package randomforestExplainer [80]. Minimal depth is a measure showing the number of nodes required to split the data into their target classes when the variable was used at a tree node. Thus, it shows how important each variable is to split the forest to achieve homogeneity among the target classes. The lower the value the higher the robustness of the variable. Only single-variable feature importance was considered. Interactions between movement and habitat parameters were not considered as the classification of the random forest according to the OOB error was already very convincing. To visualize the relationship between the predictor variables and the probability of target classes, we plotted partial dependence plots (PDPs) using the package pdp [81] for each predictor variable of the random forest model, while holding values of other variables at their respective means. The PDPs show the probability of a movement being assigned to exploratory, transient or stationary phases dependent on the values of the predictor variables.

## Results

**Fig. 2:**
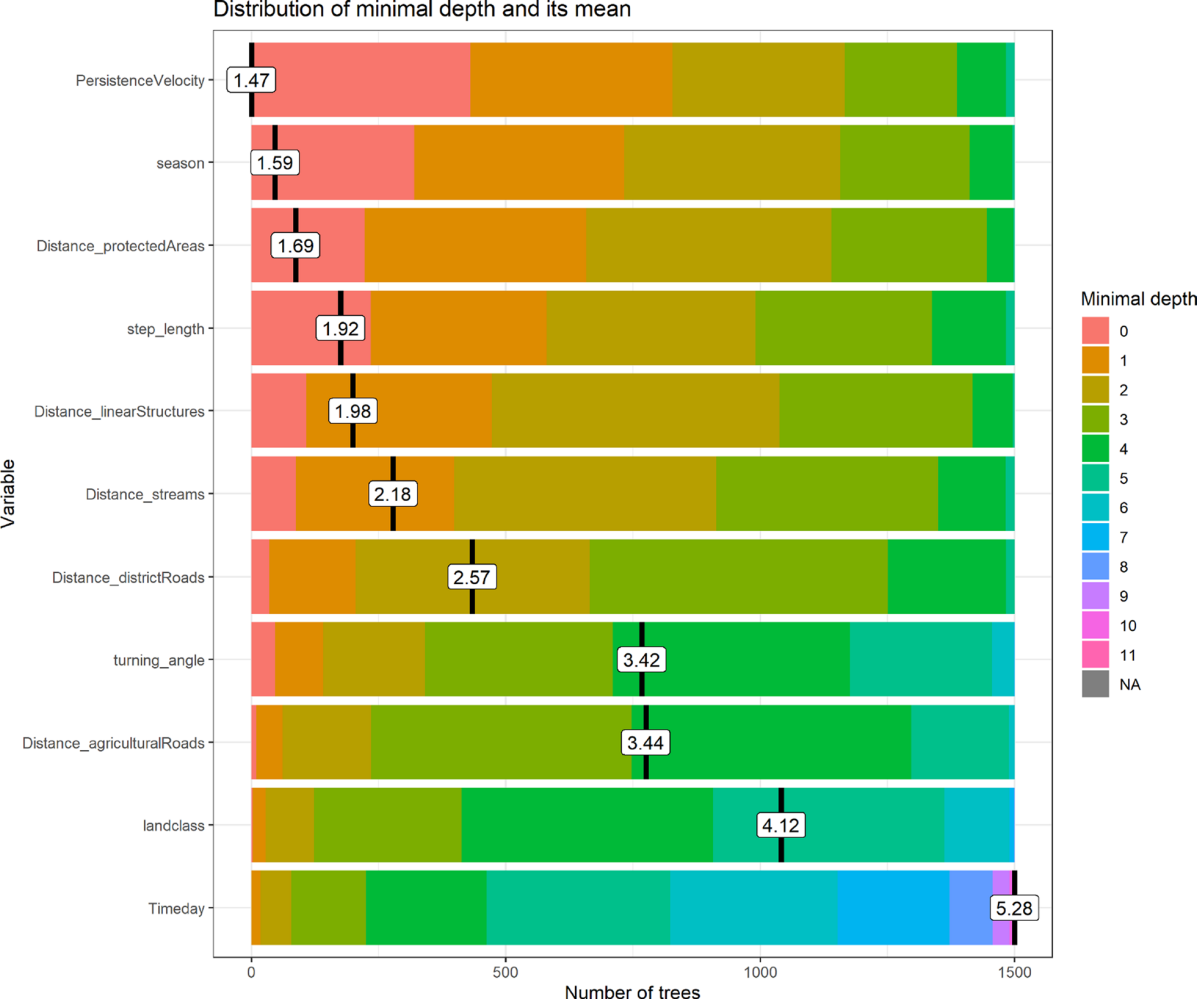
The variables of most importance for the classification of the exploratory, transient and stationary phases shown with decreasing importance. Minimal depth values indicate how important each variable is to split the given data into homogeneous classes in the random forest. The lower the value the higher the robustness of the variable. Mean of minimal depth is mean of minimal depth calculated over all the trees grown

The movement, habitat and timing parameters used as predictor variables in the random forest model classified the movement phases with 98.04% accuracy, thus the OOB estimate of error rate for the combined random forest model was 1.96 (exploratory = 1.2, transient = 0.7, and stationary = 4), see confusion matrix in Fig. 6 in the appendix for detailed information about number of observations correctly or incorrectly classified by the model for each phase. The variable ‘persistence velocity’ had the highest influence on the correct classification of the three phases, followed by the variables ‘season’, ‘distances to protected areas’, ‘step length’ and ‘distance to linear structures’ (Fig. 2).

**Fig. 3:**
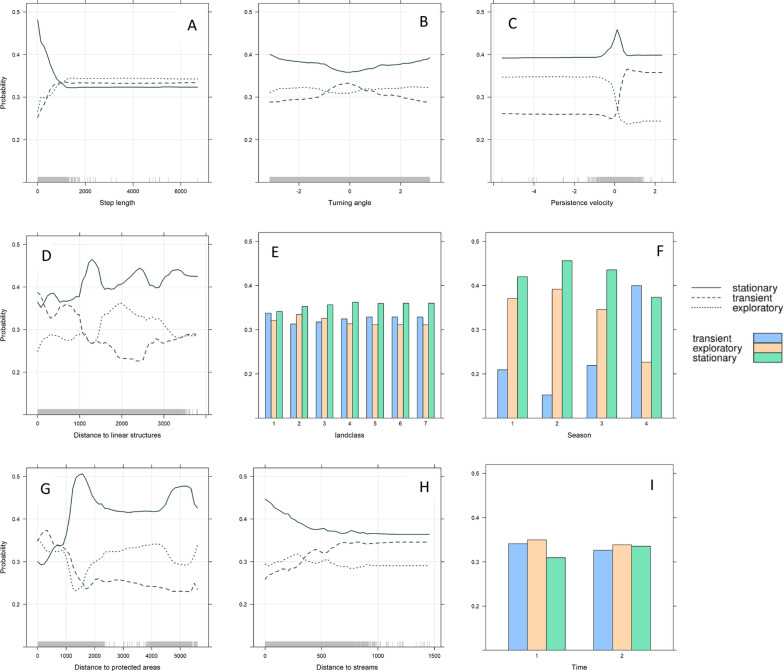
Partial dependence plots depicting the probability of three target classes, the movement phases, across the range of values of movement variables (**A**-**C**), habitat variables (**D**/**E**/**G**/**H**) and time variables (**F/I**) when other variables were kept constant. It shows the probability a location is associated to the specific target class dependent on the variables. Time: 1 = night, 2 = Day, Season: 1 = winter, 2 = spring, 3 = summer, 4 = autumn, Landclass: 1 = Tree cover, 2 = Greenland, 3 = Agricultural field, 4 = Settlement, 5 = Sparse Vegetation, 6 = Water, 7 = Wetland, Distance variables to landscape features (D/G/H) are shown in meters, as well as step length (A). Turning angle is shown in radian (B). The rugs display the data distribution. The findings for the variable Time represent the nature of the training data

**Fig. 4:**
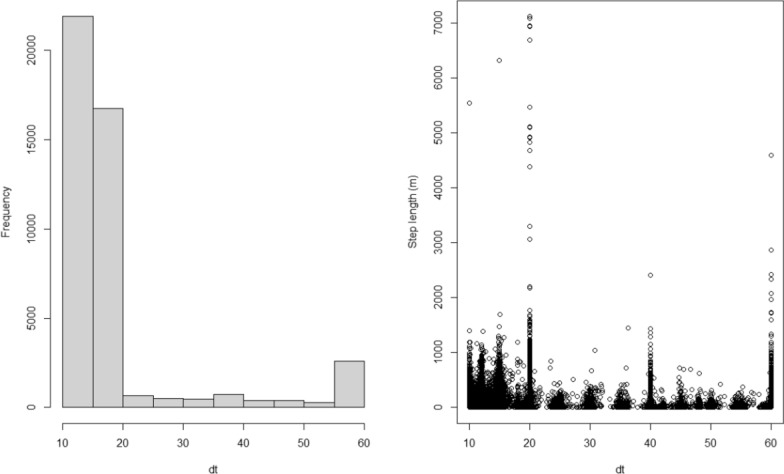
A histogram of the variable (dt) and a scatter-plot for dt ~ step length, based on the data of the dispersers (n=12). To test the association between dt and step length we computed the correlation coefficient between dt and step length for each individual. We used the Spearman correlation coefficient as both variables did not meet the assumption of normality. The local significance level is set to 0.004 asserting a global significance level of 0.05 (Bonferroni correction − 0.05/12). For four out of 12 individuals, we detected a positive, but not significant correlation (the smallest p value was equal to 0.004). We further calculated a generalized linear model analyzing the association between step length and dt. The individual was used as a random factor. The output of the model reads as: fixed effects: Estimate =  − 2.089, SE = 0.08, t = − 25.97; random effect ID: Variance = 1611, SD = 40.13, suggesting a negative association between step length and dt

**Fig. 5:**
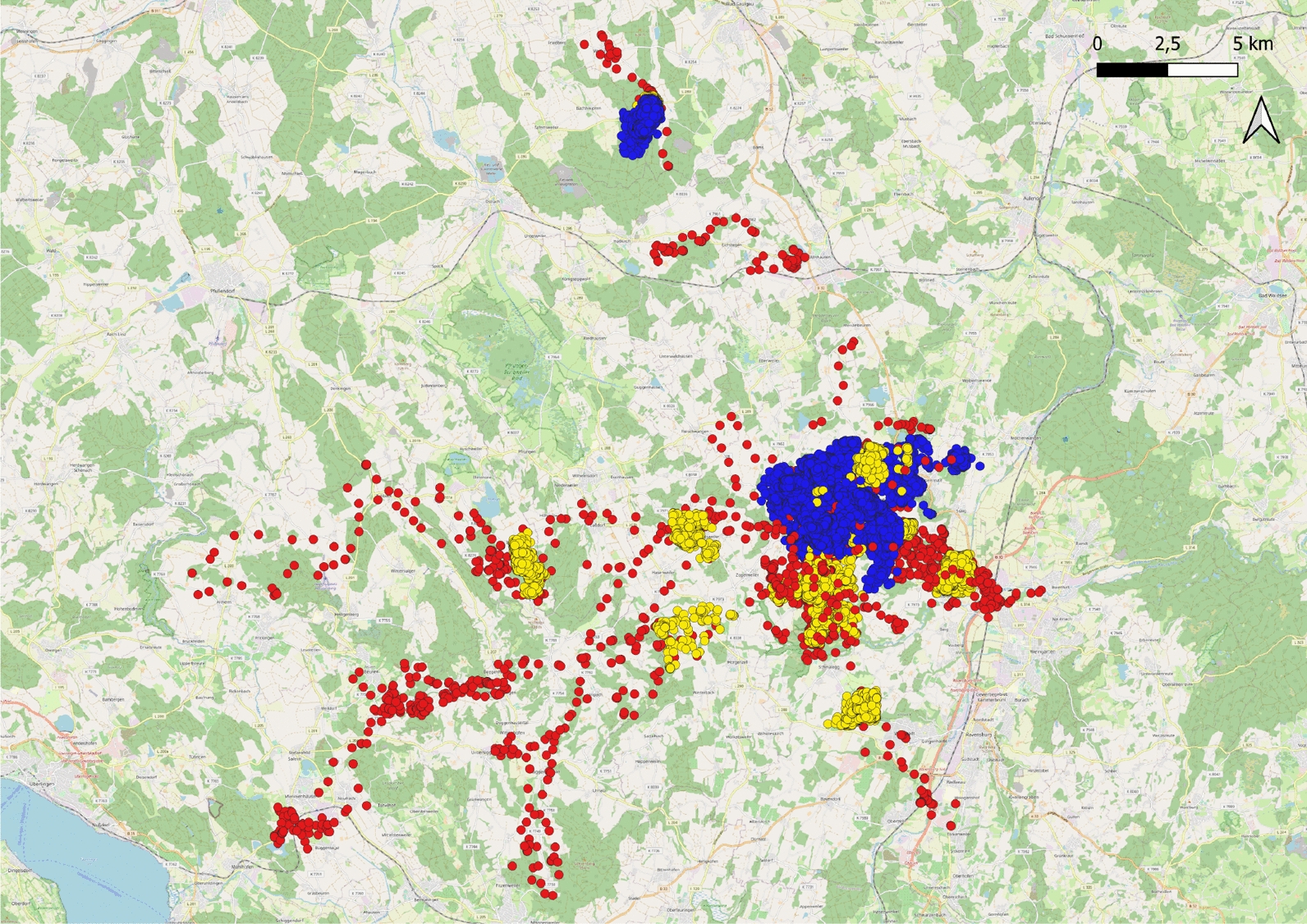
GPS points of the 12 individuals in the stationary movement phase (S_1_: blue, S_2_: yellow) and transient movement phase (red)

**Fig. 6:**
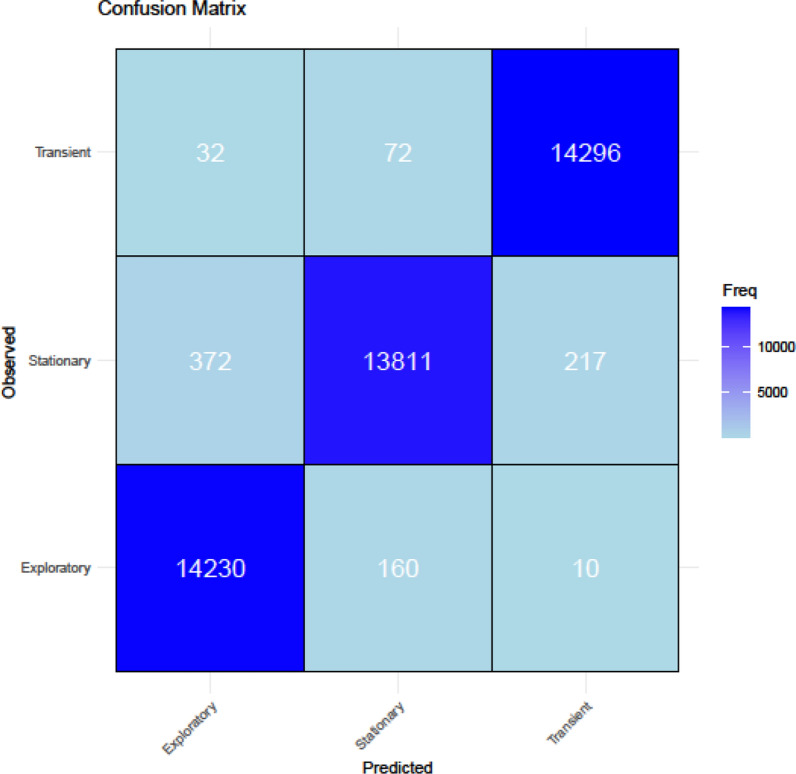
The confusion matrix graph illustrates the True Skill Statistic (TSS) and class error in model predictions, providing details on the number of observations correctly and incorrectly classified by the model for each movement phases

Looking closer at the influence of movement variables, habitat features and day-night differences on the transient movement phase, the PDPs of the random forest model show that the probability to move in the transient phase increased with a higher persistence velocity, a turning angle around 0 and higher step length (Fig. 3A–C). The probability for a GPS location to be predicted as transient increased for a lower distance to linear structures (Fig. 3D). This confirms our prediction P1. During exploratory-like movements, red fox movements were characterized by a more tortuous path resulting in a higher probability to be exploratory with decreasing persistence velocity (Fig. 3C) and a constant probability of being exploratory across all turning angles (Fig. 3B). This confirms our prediction P2 partially. Contrary to our prediction P2, the PDPs show that the probability to be exploratory increased with step length (Fig. 3A), as with transient movements. The probability to be predicted as exploratory-like movement increased with distance to linear structures until a threshold of two kilometers (Fig. 3D). Seasonal differences are shown in the PDP (Fig. 3F), where the probability of being in a transient phase doubles in autumn, and the probability of being exploratory increases in spring, which confirms our prediction P3, that seasonal differences will occur between transient and exploratory-like movements. The results of the PDP of the landclass variable show that the heterogeneous landscape is reflected in the habitat selection for all movement phases, by similar preferences of different habitat types (Fig. 3E). The probability to be exploratory or transient increased when individuals moved closer to protected areas, in comparison to stationary phase (Fig. 3G). Comparing transient and exploratory-like movements, the probability of transient movements was higher when red foxes oriented closer to protected areas. In contrast, the probability to be predicted as transient increased with distance to streams (Fig. 3H). The probability to be predicted as stationary phase increased with small step length, a variable turning angle preferred around − 2 and + 2 rad with a persistence velocity around 0 and an increase in distance to linear structures (Fig. 3A–D). The probability to be predicted as stationary did change slightly between seasons with a higher probability in spring and a lower probability in autumn (Fig. 3F).

Taking a more detailed look at the PDPs revealed a higher estimated probability for being stationary (B, C, H). In contrast to other variables, nearly equal probabilities for all three phases were observed for the variable Time which signifies no influence of the variable in determining species movement phases, as also supported by its lowest ranking observed in the variable importance graph.

## Discussion

In this study we highlighted that different movement phases of red foxes (i.e. stationary, exploratory, transient) can be described not only according to movement parameters (turning angle, step length, persistence velocity) but also according to information about habitat parameters (distance to protected areas, distance to anthropogenic linear infrastructure) and timing of the movement (season). Movement patterns of red foxes differed between transient, exploratory and stationary phases, reflecting displacement or searching behavior in extraterritorial areas and habituated, daily movements in a settled area. The variables with the highest differentiations between the phases and thus of most importance for reflecting the given classification of exploratory, transient and stationary phases were ‘persistence velocity’, ‘season’, ‘distances to protected areas’, ‘step length’ and ‘distances to linear structures’. Individually, the predictor variables had a probability of less than 50 percent to predict any of the movement phases, with the exception of the variable ‘distance to protected areas’ which had a probability greater than 50 percent for predicting a stationary phase within a distance between 1.3 and 1.6 km (Fig. 3). This signifies the importance of the combined effect of using movement and habitat parameters together that results in a very low class error rate and in the accurate classification of the movement phases [31]. Random forest models are a robust method with low generalization error, low correlation between classifier and with a high classification strength [78]. The high classification accuracy of our random forest model resulted from the balanced resampled data, where we excluded bias of uneven classes.

### Transient phase

During the transient phase, our results show that red foxes move with a high persistence velocity and an increased step length indicating continuous straight movements. This is in line with other studies focusing on dispersal movements, where movement patterns reflect a fast and straight movement to bridge long distances in a short time [20, 21]. By moving straight and fast, energy is saved and risk to encounter unpredictable events reduced [23, 82]. A study by Whittington et al. [83] revealed that carnivores increased their speed when near towns and roads. Our results indicated, that red foxes moved closer to anthropogenic linear structures during the transient phase. Distance to anthropogenic linear structures represent distances to state roads, to federal highways and to railways. Thus, moving closer to anthropogenic linear structures is likely a strategy employed that enhances fast and straight movements and thus increases displacement. Alternatively, fast movements along linear structures increases the hunting behavior and the hunting efficiency, as studied on movements of grey wolves (*Canis lupus*) [84, 85]. However, moving closer to linear structures might also be a response to the distribution of habitat features in our area, where being more active during transient movements, increases the chance of reaching or crossing linear structures.

### Exploratory phase

During the exploratory phase, movement patterns are defined by a tortuous path with decreasing persistence velocity and high variations in turning angles. This describes a searching behavior [26], which could be conducted due to no a priori knowledge about resources while exploring extraterritorial areas [26]. Contrary to our prediction P2, a higher step length was slightly more likely to be predicted as exploratory, same as transient movements. The fact that movements during exploratory phase were characterized by a rather large step length might be due to enhancing foraging and exploration efficiency by fast movements as a risk mitigation to avoid conspecifics and risky encounters [9–11] and to gather information about the surrounding [25, 29]. The probability to be predicted as stationary increased with small step lengths. This is in line with the study by Soulsbury et al. [20], where extraterritorial movements included longer distances travelled with a higher intensity of search and thoroughness than movements inside a home range.

### Space and time

Habitats including streams can represent areas with high vegetation and many shelter opportunities. Protected areas can represent a safe area with the potential to escape anthropogenic disturbances [86]. The probability to be predicted as exploratory or transient was high when individuals moved closer to protected areas, which could be due to the higher risk exposure during extraterritorial movements.

Individuals moving closer to streams had a higher probability of being predicted as stationary, which might be due to the distribution of individuals in our study area, whereas the probability of being classified as transient increased as distance to streams increased. Avoiding the territories of other, un-collared individuals, could lead to transient individuals moving in areas further from streams [24]. Peaks in the PDPs, for example the higher probability to be predicted as stationary with a distance of 1 km and 5 km away from protected areas, might reflect the landscape pattern and thus, how protected areas are distributed within our study area. Accordingly, we expect that differences of the probabilities shown in the PDPs (Fig. 3) related to distances towards habitat features rather than present species specific preferences in the vicinity. However, for large distances further away than one or more kilometers, the probabilities likely reflect also the landscape. In this context, it should be mentioned that the probabilities of each phase are relative to the probabilities in the other phases. This means that the probabilities for the three phases in the PDPs always sum to one.

Red foxes show evidence of all movement phases within all landclasses. There is a small tendency that red foxes prefer forest patches during transient phase and open areas as greenland fields during exploratory phase. However, these differences in probabilities are roughly two percent. The heterogeneous distribution of the landclasses per movement phase might result from the broad classification of the ESA satellite data within only seven categories. However, this also likely reflects the assumed spatio-temporal behavior of a generalist and emphasizes that red foxes use different landscape types in heterogeneous areas, where field sizes are small and diverse [44]. While red foxes are typically a crepuscular and nocturnal species [87], our results show that the different movement phases were independent of the time of day. Season is of high importance in distinguishing the movement phases. Transient movements mainly took place during autumn, which reflects the dispersal timing of subadult foxes [20]. Exploratory-like movements were mainly conducted during winter and spring, which might be due to mate searching and the breeding season of red foxes [20].

## Conclusion

In the presented study, we used movement data with a high temporal and spatial resolution to provide insights into how an adaptable generalist species such as the red fox explores extraterritorial areas. In the context of environmental change such as climate change, anthropogenic habitat alterations, and the depletion of natural habitat, it can be expected that an increasing number of species might have to navigate extraterritorial and completely novel areas during their lifetime [88, 89]. In the future, species communities could increasingly consist of generalists, whereas the number of specialists will decrease [90, 91], as generalists will profit from their behavioral and dietary flexibility [92–94]. Our results further contribute to a better understanding of how a generalist species, the red fox, moves in different movement phases and how these movement patterns are influenced by habitat and time variables. Furthermore, our results signify the importance of the combined effect of using movement, habitat and time variables together in analyzing movement phases. In extraterritorial areas, red foxes either move more tortuously with multiple reorientations during exploratory-like movements reflecting a searching behavior, or they focus on quick displacement by moving straight with high persistence velocity during transient movements. High movement variability may allow red foxes to navigate in extraterritorial areas efficiently and to adapt to different environmental and behavioral conditions.

## Data Availability

The datasets analyzed during the current study are not publicly available due to further analyses of an ongoing PhD project.

## Appendix

### Outlier removal

To exclude potential outliers in our dataset, we first removed GPS points which received less than four satellites [95] (Fig. 4). Second, we removed all GPS points that were not located in the federal district of Oberschwaben, since the collared red foxes stayed inside the region Oberschwaben (see appendix Fig. 5). Thus, all points outside the district could be treated easily as outliers. Third, when two subsequent locations have had the same timestamp or were located at identical coordinates we only kept the first location for further analysis. Additionally, we removed the points where the Euclidian distance between two consecutive points (d(x[t], x[t + 1])) was larger than the 99.5% of the quantile of all calculated Euclidian distances with the additional condition that the distance d(x[t], x[t + 2]) was less than 1 km (Tables 2 and 3, Fig. 6).

**Table 2:** Study animals with Fox ID, age, weight, collar type with different data collection frequencies, start date and end date of data collection, total number of days collecting data, final total number of GPS points. Bold individuals are the twelve individuals used in this data analysis

Fox ID	Age	Weight [kg]	Collar Type	Start date	End date	No. days	No. GPS points
*Male*							
**FM1**	**Adult**	6.1	**e-obs**	**2020–11-07**	**2021–07-16**	**251**	**12,661**
**FM2**	**Adult**	6.5	**e-obs**	**2020–12-17**	**2021–01-30**	**44**	**2692**
FM3	Subadult	5	Followit	2021–09-08	2021–09-24	15	166
**FM4**	**Subadult**	5.1	**Followit**	**2021–09-09**	**2021–11-22**	**74**	**1899**
**FM5**	**Subadult**	5.6	**Followit**	**2021–09-10**	**2021–12-20**	**101**	**3334**
**FM6**	**Subadult**	5.3	**Followit**	**2021–09-27**	**2021–12-23**	**87**	**2855**
FM7	Subadult	5.5	Followit	2021–10-03	2021–11-30	57	2453
**FM8**	**Subadult**	6.5	**e-obs**	**2021–10-16**	**2021–11-12**	**27**	**1113**
**FM9**	**Subadult**	6.5	**Followit**	**2021–10-19**	**2021–12-09**	**51**	**2095**
**FM10**	**Subadult**	7.1	**Followit**	**2021–10-31**	**2022–01-23**	**84**	**3004**
FM11	Subadult	6	e-obs	2021–12-24	2022–09-21	271	9402
FM12	Adult	5	e-obs	2022–01-17	2022–09-21	247	9873
FM13	Adult	6	e-obs	2022–01-25	2022–09-23	241	11,084
FM14	Subadult	6.5	Followit	2022–10-04	2022–12-16	73	2784
FM15	Subadult	7	Followit	2022–10-20	2023–01-19	91	3558
FM16	Subadult	5.7	Followit	2022–10-24	2023–01-11	79	2698
**FM17**	**Subadult**	6	**Followit**	**2022–11-10**	**2023–01-11**	**62**	**2346**
FM18	subadult	5.7	e-obs	2022–12-08	2023–01-06	29	693
**FM19**	**Subadult**	6.5	**e-obs**	**2023–01-21**	**2023–03-23**	**61**	**2870**
*Female*							
**FF1**	**Subadult**	5.6	**e-obs**	**2020–10-29**	**2021–01-30**	**93**	**4487**
FF2	Adult	4.9	e-obs	2021–01-01	2021–01-12	11	359
FF3	Subadult	5.1	e-obs	2021–01-15	2021–07-27	193	10,584
**FF4**	**Subadult**	4.9	**e-obs**	**2021–11-04**	**2022–04-20**	**167**	**6980**
FF5	Subadult	6.7	e-obs	2021–12-22	2022–09-28	280	11,937
FF6	Adult	6.5	e-obs	2022–01-09	2022–07-08	180	7254
FF8	Subadult	5.5	Followit	2022–11-28	2023–01-15	48	1703

**Table 3:** Number of GPS points per habitat class (ESA land classification)

Habitat classes	1	2	3	4	5/6/7
GPS points	55,885	87,189	71,727	10,753	110

1, Tree cover; 2, Greenland; 3, Agricultural field; 4, Settlement; 5, Sparse Vegetation; 6, Water; 7, Wetland

### Classification of exploratory movements

During the current research, we classified GPS locations of collared red foxes as exploratory, which were initially classified as stationary by Oehler et al. [68]. Therefore, we applied an individual based criteria based on both the distribution of coordinates (x, y) and the distribution of the net squared displacement (NSD) values. In case either the x-coordinate, the y-coordinate or the value of the NSD was smaller (bigger) than the 1.5% (98.5%) quantile of the corresponding stationary period, the GPS location was labelled as exploratory. Since this definition would always lead to exploratory movements even for very stationary individuals with a relatively small home range we added a further pre-condition that for each location labelled as exploratory the distance towards the first location should be bigger than 1 km.

The choice for these quantiles were based on visual inspection of both “the NSD-plot” (right column in Tables 4 and 5) and the plot of the “movement trajectory” (left column in Tables 4 and 5). We increased or decreased the threshold until values led to a classification of exploratory movements that met our expectations. During this procedure, we noticed that a classification based only on x and y coordinates or only on the NSD-value led to rather unsatisfactory classifications. Thus, we combined both conditions. We increased and decreased the threshold for the two sided quantiles (Table 5). The two sided quantile of 1.5% led to the most reasonable classification of exploratory movements for all individuals.

To better understand the consequences of (a) using the coordinates or the NSD value as potential criteria for exploratory movements and (b) the choice of quantile-threshold value (here a two sided value of 1.5%) we provide some examples in Table 4 and 5. All subsequent plots show either the movement trajectory or the NSD-value (normed to kilometers) of the individual shown in the main manuscript.

**Table 4:** Labelling exploratory points (green) according to coordinates, the NSD or both criteria. Blue points show the first stationary phase, yellow points show the second stationary phase, green points show the exploratory points, red points show the transient phase

Quantile two sided	Movement trajectory (x–y Plot)	Net squared displacement (NSD)
1.5 / 98.5 (only for coordinates)		
**1.5 / 98.5** (both criteria applied [logical “or”])		
1.5 / 98.5 (only for nsd)

**Table 5:** Labelling exploratory points (green) using different thresholds for the quantiles. Blue points show the first stationary phase, yellow points show the second stationary phase, green points show the exploratory points, red points show the transient phase

Quantile two sided	Movement trajectory (x–y Plot)	Net squared displacement (NSD)
0.5 / 99.5 (both criteria applied [logical “or”])		
**1.5 / 98.5** (both criteria applied [logical “or”])		
2.5 / 97.5 (both criteria applied [logical “or”])		

## Abbreviations

GPS: Global positioning system
OOB: Out of bag
TA: Turning angle
SL: Step length
PV: Persistence velocity
PDP: Partial dependence plot

## References

[1] Fryxell JM, Sinclair ARE. Causes and consequences of migration by large herbivores. Trends Ecol Evol. 1988;3(9):237–41. 21227239 10.1016/0169-5347(88)90166-8

[2] Spencer WD. Home ranges and the value of spatial information. J Mammal. 2012;93(4):929–47.

[3] Wetterer JK. Central place foraging theory: when load size affects travel time. Theor Popul Biol. 1989;36(3):267–80.

[4] Fleming CH, Calabrese JM, Mueller T, Olson KA, Leimgruber P, Fagan WF. From fine-scale foraging to home ranges: a semivariance approach to identifying movement modes across spatiotemporal scales. Am Nat. 2014;183(5):E154–67. 24739204 10.1086/675504

[5] Lima SL, Zollner PA. Towards a behavioral ecology of ecological landscapes. Trends Ecol Evol. 1996;11(3):131–5. 21237783 10.1016/0169-5347(96)81094-9

[6] Guilford T, Biro D. Route following and the pigeon’s familiar area map. J Exp Biol. 2014;217(2):169–79. 24431141 10.1242/jeb.092908

[7] Sawyer H, Merkle JA, Middleton AD, Dwinnell SPH, Monteith KL. Migratory plasticity is not ubiquitous among large herbivores. J Anim Ecol. 2019;88(3):450–60. 30449042 10.1111/1365-2656.12926

[8] Roshier DA, Signer J, Carter A. Visitation of artificial watering points by the red fox (Vulpes vulpes) in semiarid Australia. Ecol Evol. 2021;11(14):9815–26. 34306664 10.1002/ece3.7810 PMC8293723

[9] Bonnet X, Naulleau G, Shine R. The dangers of leaving home: dispersal and mortality in snakes. Biol Cons. 1999;89(1):39–50.

[10] Neumann W, Ericsson G, Dettki H, Bunnefeld N, Keuler NS, Helmers DP, et al. Difference in spatiotemporal patterns of wildlife road-crossings and wildlife-vehicle collisions. Biol Cons. 2012;145(1):70–8.

[11] Gese EM. Territorial defense by coyotes (Canis latrans) in Yellowstone National Park, Wyoming: who, how, where, when, and why. Can J Zool. 2001;79(6):980–7.

[12] Yoder JM, Marschall EA, Swanson DA. The cost of dispersal: predation as a function of movement and site familiarity in ruffed grouse. Behav Ecol. 2004;15(3):469–76.

[13] Bonte D, Van Dyck H, Bullock JM, Coulon A, Delgado M, Gibbs M, et al. Costs of dispersal. Biol Rev. 2012;87(2):290–312. 21929715 10.1111/j.1469-185X.2011.00201.x

[14] Schmidt KA, Dall SR, Van Gils JAJO. The ecology of information: an overview on the ecological significance of making informed decisions. Oikos. 2010;119(2):304–16.

[15] Berger-Tal O, Nathan J, Meron E, Saltz D. The exploration-exploitation dilemma: a multidisciplinary framework. PLoS ONE. 2014;9(4): e95693. 24756026 10.1371/journal.pone.0095693 PMC3995763

[16] Bowler DE, Benton TG. Causes and consequences of animal dispersal strategies: relating individual behaviour to spatial dynamics. Biol Rev. 2005;80(2):205–25. 15921049 10.1017/s1464793104006645

[17] Bell WJ. Searching behavior patterns in insects. Annu Rev Entomol. 1990;35(1):447–67.

[18] Fagan WF, Lewis MA, Auger-Méthé M, Avgar T, Benhamou S, Breed G, et al. Spatial memory and animal movement. Ecol Lett. 2013;16(10):1316–29. 23953128 10.1111/ele.12165

[19] Jonsen ID, Taylor PD. Fine-scale movement behaviors of calopterygid damselflies are influenced by landscape structure: an experimental manipulation. Oikos. 2000;88(3):553–62.

[20] Soulsbury CD, Iossa G, Baker PJ, White PCL, Harris S. Behavioral and spatial analysis of extraterritorial movements in red foxes (Vulpes vulpes). J Mammal. 2011;92(1):190–9.

[21] Van Dyck H, Baguette M. Dispersal behaviour in fragmented landscapes: routine or special movements? Basic Appl Ecol. 2005;6(6):535–45.

[22] Mate BR, Gisiner R, Mobley J. Local and migratory movements of Hawaiian humpback whales tracked by satellite telemetry. Can J Zool. 1998;76(5):863–8.

[23] Klarevas-Irby JA, Wikelski M, Farine DR. Efficient movement strategies mitigate the energetic cost of dispersal. Ecol Lett. 2021;24(7):1432–42. 33977638 10.1111/ele.13763

[24] Arnold J, Soulsbury CD, Harris S. Spatial and behavioral changes by red foxes (Vulpes vulpes) in response to artificial territory intrusion. Can J Zool. 2011;89(9):808–15.

[25] Bartumeus F, Campos D, Ryu WS, Lloret-Cabot R, Méndez V, Catalan J. Foraging success under uncertainty: search tradeoffs and optimal space use. Ecol Lett. 2016;19(11):1299–313. 27634051 10.1111/ele.12660

[26] Bartumeus F, da Luz MGE, Viswanathan GM, Catalan J. Animal search strategies: a quantitative random-walk analysis. Ecology. 2005;86(11):3078–87.

[27] Dorfman A, Hills TT, Scharf I. A guide to area-restricted search: a foundational foraging behaviour. Biol Rev. 2022;97(6):2076–89. 35821610 10.1111/brv.12883 PMC9796321

[28] Bennetts RE, Kitchens WM. Factors influencing movement probabilities of a nomadic food specialist: proximate foraging benefits or ultimate gains from exploration? 2000;91(3):459–67.

[29] Pisula W, Modlinska KJAB, Cognition. Animals in search of stimulation and information: a review of over 10 years of our research on spontaneous exploration in rats as a response to novelty in low-stress paradigm. 2023;10(4):287–303.

[30] Maresh Nelson SB, Coon JJ, Miller JR. Do habitat preferences improve fitness? Context-specific adaptive habitat selection by a grassland songbird. Oecologia. 2020;193(1):15–26. 32201907 10.1007/s00442-020-04626-8

[31] Nathan R, Getz WM, Revilla E, Holyoak M, Kadmon R, Saltz D, et al. A movement ecology paradigm for unifying organismal movement research. Proc Natl Acad Sci. 2008;105(49):19052–9. 19060196 10.1073/pnas.0800375105 PMC2614714

[32] Mayor SJ, Schneider DC, Schaefer JA, Mahoney SP. Habitat selection at multiple scales. Écoscience. 2009;16(2):238–47.

[33] Matthiopoulos J, Fieberg J, Aarts G, Beyer HL, Morales JM, Haydon DT. Establishing the link between habitat selection and animal population dynamics. Ecol Monogr. 2015;85(3):413–36.

[34] Northrup JM, Vander Wal E, Bonar M, Fieberg J, Laforge MP, Leclerc M, et al. Conceptual and methodological advances in habitat-selection modeling: guidelines for ecology and evolution. Ecol Appl. 2022;32(1): e02470. 34626518 10.1002/eap.2470 PMC9285351

[35] Nisi AC, Suraci JP, Ranc N, Frank LG, Oriol-Cotterill A, Ekwanga S, et al. Temporal scale of habitat selection for large carnivores: balancing energetics, risk and finding prey. J Anim Ecol. 2022;91(1):182–95. 34668571 10.1111/1365-2656.13613 PMC9298125

[36] Thorsen NH, Hansen JE, Støen OG, Kindberg J, Zedrosser A, Frank SC. Movement and habitat selection of a large carnivore in response to human infrastructure differs by life stage. Mov Ecol. 2022;10(1):52. 36447280 10.1186/s40462-022-00349-y PMC9706841

[37] Salvatori M, De Groeve J, van Loon E, De Baets B, Morellet N, Focardi S, et al. Day versus night use of forest by red and roe deer as determined by corine land cover and copernicus tree cover density: assessing use of geographic layers in movement ecology. Landscape Ecol. 2022;37(5):1453–68.

[38] Richter L, Balkenhol N, Raab C, Reinecke H, Meißner M, Herzog S, et al. So close and yet so different: the importance of considering temporal dynamics to understand habitat selection. Basic Appl Ecol. 2020;43:99–109.

[39] Birkett PJ, Vanak AT, Muggeo VMR, Ferreira SM, Slotow R. Animal perception of seasonal thresholds: changes in elephant movement in relation to rainfall patterns. PLoS ONE. 2012;7(6): e38363. 22761680 10.1371/journal.pone.0038363 PMC3384670

[40] Lewis JS, Rachlow JL. Activity patterns of black bears in relation to sex, season, and daily movement rates. Western North Am Nat. 2011;71(3):388–95.

[41] Larivière S, Pasitschniak-Arts M. Vulpes vulpes. Mamm Species. 1996;537:1–11.

[42] Elmhagen B, Berteaux D, Burgess RM, Ehrich D, Gallant D, Henttonen H, et al. Homage to Hersteinsson and Macdonald: climate warming and resource subsidies cause red fox range expansion and Arctic fox decline. Polar Res. 2017;36(sup1):3.

[43] Soulsbury CD, Statham MJ. Red fox Vulpes vulpes Linnaeus. In: Hackländer K, Zachos FE, editors. Handbook of the mammals of Europe. Cham: Springer; 1758.

[44] Cagnacci F, Meriggi A, Lovari S. Habitat selection by the red fox *Vulpes vulpes* (L. 1758) in an Alpine area. Ethol Ecol Evol. 2004;16(2):103–16.

[45] Cavallini P, Lovari S. Home range, habitat selection and activity of the red fox in a Mediterranean coastal ecotone. Acta Theriol. 1994;39(3):279–87.

[46] Storm GL, Andrews RD, Phillips RL, Bishop RA, Siniff DB, Tester JR. Morphology, reproduction, dispersal, and mortality of midwestern red fox populations. Wildl Monogr. 1976;49:3–82.

[47] Bischof R, Gjevestad JGO, Ordiz A, Eldegard K, Milleret C. High frequency GPS bursts and path-level analysis reveal linear feature tracking by red foxes. Sci Rep. 2019;9(1):8849. 31221989 10.1038/s41598-019-45150-x PMC6586955

[48] Wolfe A, Hogan S, Maguire D, Fitzpatrick C, Mulcahy G, Vaughan L, et al. Red foxes (*Vulpes vulpes*) in Ireland as hosts for parasites of potential zoonotic and veterinary significance. Vet Rec. 2001;149(25):759–63. 11808662

[49] Tryjanowski P, Gołdyn B, Surmacki A. Influence of the red fox (*Vulpes vulpes*, Linnaeus 1758) on the distribution and number of breeding birds in an intensively used farmland. Ecol Res. 2002;17(3):395–9.

[50] Gol B, Hromada M, Surmacki A, Tryjanowski P. Habitat use and diet of the red fox *Vulpes vulpes* in an agricultural landscape in Poland. Z Jagdwiss. 2003;49:1–10.

[51] Bundesamt für Naturschutz:Landschaftssteckbrief Oberschwäbisches Hügelland. de-DE [Internet]. 2010 [cited 22.01.2023]. Available from: https://www.bfn.de/landschaftssteckbriefe/oberschwaebischeshuegelland.

[52] Statistisches Landesamt B-W. Landwirtschaftszählung 2020 – Viehhaltung im Land 2021; Statistik Aktuell.

[53] Rieke J, Wöllper F. Flächen für Landwirtschaft in den Kreisen Baden-Württembergs. 2018; Statistisches Monatsheft Baden-Württemberg(9/2018).

[54] Klimadaten in den Gemeinden Baden-Württembergs. de-DE [Internet]. Landesanstalt für Landwirtschaft, Ernährung und Ländlichen Raum. 2019 [cited 22.01.2023]. Available from: https://www.lel-web.de/app/ds/lel/a3/Online_Kartendienst_extern/Karten/92411/index.html.

[55] Shilo Y, Lapid R, King R, Bdolah-Abram T, Epstein A. Immobilization of red fox (*Vulpes vulpes*) with medetomidine-ketamine or medetomidine-midazolam and antagonism with atipamezole. J Zoo Wildl Med. 2010;41(1):28–34. 20722251 10.1638/2009-0028.1

[56] Harris S. Age determination in the Red fox (*Vulpes vulpes*)—an evaluation of technique efficiency as applied to a sample of suburban foxes. J Zool. 1978;184(1):91–117.

[57] Cavallini P, Santini S. Age determination in the red fox in a Mediterranean habitat. Zeitschrift fur Saugetierkunde. 1995;60:136.

[58] Roulichová J, Anděra M. Age determination in the Red Fox (*Vulpes vulpes*): a comparative study. Lynx. 2007;38:55–71.

[59] Chevallier C, Gauthier G, Berteaux D. Age estimation of live arctic foxes *Vulpes lagopus* based on teeth condition. Wildlife Biol. 2017;2017(1):00304.

[60] Team QD. QGIS geographic information system. QGIS Association; 2023.

[61] Brivio F, Grignolio S, Sica N, Cerise S, Bassano B. Assessing the impact of capture on wild animals: the case study of chemical immobilisation on alpine ibex. PLoS ONE. 2015;10(6): e0130957. 26111118 10.1371/journal.pone.0130957 PMC4482404

[62] Signer J, Fieberg J, Avgar T. Animal movement tools (amt): R package for managing tracking data and conducting habitat selection analyses. Ecol Evol. 2019;9(2):880–90. 30766677 10.1002/ece3.4823 PMC6362447

[63] Team RC. R: A language and environment for statistical computing. R Foundation for Statistical Computing. 2022.

[64] Bastille-Rousseau G, Potts JR, Yackulic CB, Frair JL, Ellington EH, Blake S. Flexible characterization of animal movement pattern using net squared displacement and a latent state model. Mov Ecol. 2016;4(1):15. 27252856 10.1186/s40462-016-0080-y PMC4888472

[65] Spitz DB, Hebblewhite M, Stephenson TR. ‘MigrateR’: extending model-driven methods for classifying and quantifying animal movement behavior. 2017;40(6):788–99.

[66] Gurarie E, Cagnacci F, Peters W, Fleming CH, Calabrese JM, Mueller T, et al. A framework for modelling range shifts and migrations: asking when, whither, whether and will it return. 2017;86(4):943–59. 10.1111/1365-2656.12674 28369891

[67] Edelhoff H, Signer J, Balkenhol N. Path segmentation for beginners: an overview of current methods for detecting changes in animal movement patterns. Mov Ecol. 2016;4(1):21. 27595001 10.1186/s40462-016-0086-5 PMC5010771

[68] Oehler F, Arnold J, Hackländer K, Signer J, Schai-Braun SC, Hagen R. Identifying dispersal events of red foxes (*Vulpes vulpes*) using early warning signals. Mov Ecol. 2024 (in Review). 10.1186/s40462-025-00579-w PMC12309036 40731370

[69] Scheffer M, Bascompte J, Brock WA, Brovkin V, Carpenter SR, Dakos V, et al. Early-warning signals for critical transitions. Nature. 2009;461(7260):53–9. 19727193 10.1038/nature08227

[70] Dakos V, Carpenter SR, Brock WA, Ellison AM, Guttal V, Ives AR, et al. Methods for detecting early warnings of critical transitions in time series illustrated using simulated ecological data. PLoS ONE. 2012;7(7): e41010. 22815897 10.1371/journal.pone.0041010 PMC3398887

[71] Börger L, Fryxell JJDe, Evolution. Quantifying individual differences in dispersal using net squared displacement. 2012;30:222-30

[72] Gurarie E, Cheraghi FJRpv-. marcher: migration and range change estimation in R. 2017.

[73] Gurarie E, Andrews RD, Laidre KL. A novel method for identifying behavioural changes in animal movement data. Ecol Lett. 2009;12(5):395–408. 19379134 10.1111/j.1461-0248.2009.01293.x

[74] Gurarie E, Bracis C, Delgado M, Meckley TD, Kojola I, Wagner CM. What is the animal doing? Tools for exploring behavioural structure in animal movements. J Anim Ecol. 2016;85(1):69–84. 25907267 10.1111/1365-2656.12379

[75] Zanaga D, Van De Kerchove R, Daems D, De Keersmaecker W, Brockmann C, Kirches G, Wevers J, Cartus O, Santoro M, Fritz S, Lesiv M, Herold M, Tsendbazar N-E, Xu P, Ramoino F, Arino O. ESA WorldCover 10 m 2021 v200 v200 ed. Zenodo; 2022.

[76] Geofabrik. Geofabrik download server. 2023.

[77] Kuckartz U, Rädiker S, Ebert T, Schehl J. Statistik: eine verständliche Einführung. Berlin: Springer; 2013.

[78] Breiman L. Random forests. Mach Learn. 2001;45(1):5–32.

[79] Marchese Robinson RL, Palczewska A, Palczewski J, Kidley N. Comparison of the predictive performance and interpretability of random forest and linear models on benchmark data sets. J Chem Inf Model. 2017;57(8):1773–92. 28715209 10.1021/acs.jcim.6b00753

[80] Paluszynska A, Biecek P, Jiang Y, Jiang M. Package ‘randomForestExplainer’. Explaining and visualizing random forests in terms of variable importance. 2017.

[81] Greenwell BM. pdp: an R package for constructing partial dependence plots. R J. 2017;9(1):421.

[82] Roshier DA, Carter A. Space use and interactions of two introduced mesopredators, European red fox and feral cat, in an arid landscape. Ecosphere. 2021;12(7): e03628.

[83] Whittington J, Hebblewhite M, Baron RW, Ford AT, Paczkowski J. Towns and trails drive carnivore movement behaviour, resource selection, and connectivity. Mov Ecol. 2022;10(1):17. 35395833 10.1186/s40462-022-00318-5 PMC8994267

[84] Dickie M, Serrouya R, McNay RS, Boutin SJJoAE. Faster and farther: wolf movement on linear features and implications for hunting behaviour. 2017;54(1):253-63.

[85] Dickie M, McNay SR, Sutherland GD, Cody M, Avgar TJJOAE. Corridors or risk? Movement along, and use of, linear features varies predictably among large mammal predator and prey species. 2020;89(2):623–34. 10.1111/1365-2656.13130 PMC7028095 31648375

[86] Smith AF, Ciuti S, Shamovich D, Fenchuk V, Zimmermann B, Heurich M. Quiet islands in a world of fear: wolves seek core zones of protected areas to escape human disturbance. Biol Cons. 2022;276: 109811.

[87] Díaz-Ruiz F, Caro J, Delibes-Mateos M, Arroyo B, Ferreras P. Drivers of red fox (*Vulpes vulpes*) daily activity: prey availability, human disturbance or habitat structure? J Zool. 2016;298(2):128–38.

[88] Williams JE, Blois JL. Range shifts in response to past and future climate change: can climate velocities and species’ dispersal capabilities explain variation in mammalian range shifts? J Biogeogr. 2018;45(9):2175–89.

[89] Sattar Q, Maqbool ME, Ehsan R, Akhtar S, Sattar Q, Maqbool ME, et al. Review on climate change and its effect on wildlife and ecosystem. Open J Environ Biol. 2021;6(1):008–14.

[90] Lurgi M, López BC, Montoya JM. Novel communities from climate change. Philos Trans R Soc Lond B Biol Sci. 2012;367(1605):2913–22. 23007079 10.1098/rstb.2012.0238 PMC3479747

[91] Sweeney CP, Jarzyna MA. Assessing the synergistic effects of land use and climate change on terrestrial biodiversity: are generalists always the winners? Curr Landsc Ecol Rep. 2022;7(4):41–8.

[92] Elmhagen B, Kindberg J, Hellström P, Angerbjörn A. A boreal invasion in response to climate change? Range shifts and community effects in the borderland between forest and tundra. Ambio. 2015;44(1):39–50. 10.1007/s13280-014-0606-8 PMC4289007 25576279

[93] Nater CR, Eide NE, Pedersen ÅØ, Yoccoz NG, Fuglei E. Contributions from terrestrial and marine resources stabilize predator populations in a rapidly changing climate. Ecosphere. 2021;12(6): e03546.

[94] Rather TA, Kumar S, Khan JA. Multi-scale habitat selection and impacts of climate change on the distribution of four sympatric meso-carnivores using random forest algorithm. Ecol Process. 2020;9(1):60.

[95] Huang J-Y, Tsai C-H. Improve GPS positioning accuracy with context awareness. In: 2008 First IEEE International Conference on Ubi-Media Computing, Lanzhou. 2008. pp. 94-99. 10.1109/UMEDIA.2008.4570872.

